# Self-control, smartphone addiction, negative attentional bias and insomnia symptoms among college students: a latent profile and network analysis

**DOI:** 10.3389/fpsyg.2026.1976921

**Published:** 2026-10-14

**Authors:** Xiaoming Zhang, Hanbin He, Bifa Zhu, Xinhong Zhu

**Affiliations:** 1 School of Nursing, Hubei University of Chinese Medicine, Wuhan, Hubei, China; 2 School of Marxism, Hubei University of Chinese Medicine, Wuhan, Hubei, China; 3 Hubei Shizhen Laboratory, Wuhan, Hubei, China

**Keywords:** college students, insomnia symptoms, negative attentional bias, self-control, smartphone addiction

## Abstract

**Background:**

Insomnia symptoms are common among college students and may be associated with multiple psychological and behavioral characteristics. However, the relationships among self-control, smartphone addiction (SA), negative attentional bias, and insomnia symptoms remain unclear. This study aimed to identify latent profiles of self-control, SA, and negative attentional bias and further explore their network relationships with insomnia symptoms among college students.

**Methods:**

A cross-sectional survey was conducted among 1,597 Chinese college students. Latent profile analysis (LPA) was used to identify profiles based on self-control, SA, and negative attentional bias. Multivariable linear regression examined the associations between latent profile membership and Athens Insomnia Scale (AIS) scores. Network analysis was performed to identify central and bridge nodes, and the network comparison test (NCT) was used to compare network structures across latent profiles.

**Results:**

Three latent profiles were identified: the Moderate Self-Control–Moderate SA and Negative Attention Profile (75.20%), the Low Self-Control–High SA and Negative Attention Profile (10.83%), and the High Self-Control–Low SA and Negative Attention Profile (13.96%). Compared with the High Self-Control–Low SA and Negative Attention Profile, the Moderate Self-Control–Moderate SA and Negative Attention Profile (*B* = 2.31, 95% CI: 1.75–2.86) and the Low Self-Control–High SA and Negative Attention Profile (*B* = 4.04, 95% CI: 3.27–4.81) were significantly associated with higher AIS scores (both *p* < 0.001). SA1 (Withdrawal behavior) had the highest expected influence (EI) in the overall network and across all three profiles, and also exhibited the highest bridge strength in the overall network. APNI-N (Negative attention) showed the second-highest bridge strength. After false discovery rate correction, the NCT showed no significant differences in either overall network structure or global strength across the three profiles.

**Conclusion:**

Three profiles of self-control, SA, and negative attentional bias were identified among college students and showed different associations with insomnia symptoms. Withdrawal behavior and negative attention emerged as important features of the psychological and behavioral network. These findings provide further insight into the joint patterns and interconnected characteristics associated with insomnia symptoms among college students.

## 1 Introduction

Sleep is an essential process for maintaining normal physiological functioning and plays an important role in physical and mental health, learning, and daily life of college students. Insomnia is a common sleep problem characterized by difficulty falling asleep, difficulty maintaining sleep, or early morning awakening with an inability to return to sleep (Buysse, 2013). College students experience substantial changes in their lifestyles and social environments. Changes in living environments, academic and psychosocial stress, and irregular daily routines and sleep patterns may increase their likelihood of experiencing insomnia symptoms (Spyridonidis et al., 2025). A recent systematic review and meta-analysis reported a pooled prevalence of insomnia symptoms of 46.9% among undergraduate students (Spyridonidis et al., 2025), indicating that insomnia symptoms are an important health concern in this population. Previous studies have shown that insomnia symptoms are closely associated with mental health problems such as depression, anxiety, and stress, as well as poorer cognitive function and academic performance (Gardani et al., 2022; Li et al., 2024; Xu et al., 2025; Zhou et al., 2026). Therefore, identifying potential psychological and behavioral factors associated with insomnia symptoms may help promote sleep health among college students.

With the widespread use of smartphones, they have become an essential part of college students’ daily lives and are widely used for learning, social interaction, and entertainment (Sapci et al., 2021). However, excessive or uncontrolled smartphone use may also lead to a range of concerns (Li et al., 2020). Smartphone addiction (SA) is defined as underestimating the amount of time spent using a smartphone or having difficulty controlling its use, resulting in negative consequences in daily life (Leow et al., 2023). A meta-analysis involving 129,996 Chinese college students reported a pooled prevalence of SA of 28.46% (Wang and Abdullah, 2026). Previous studies have shown that SA is associated with higher levels of anxiety, depression, and impulsivity among college students (Li et al., 2020; Nikolic et al., 2023). In addition, SA is closely associated with sleep problems, with higher levels of SA being associated with poorer sleep quality and more severe insomnia symptoms (Failoc-Rojas et al., 2025; Liu et al., 2022; Nikolic et al., 2023). This association may involve both behavioral and physiological mechanisms. College students with SA may have difficulty controlling smartphone use before bedtime, which may delay bedtime and sleep onset and reduce actual sleep duration. Meanwhile, exposure to light from electronic screens before bedtime may affect melatonin secretion and delay circadian rhythms, further interfering with sleep onset (Chang et al., 2015). Longitudinal research has also found that problematic smartphone use can predict subsequent bedtime procrastination and sleep quality among college students, suggesting that problematic smartphone use may be closely related to long-term changes in sleep behaviors and sleep conditions (Cemei et al., 2024). These findings indicate a close relationship between smartphone use and sleep among college students, and the ability to regulate and control smartphone use may be an important factor in understanding this relationship.

The regulation of smartphone use may be closely related to an individual’s self-control. Self-control is defined as the ability to actively regulate one’s thoughts, emotions, and behaviors when long-term valued goals conflict with temporarily more rewarding goals (Ling et al., 2024). For college students, greater independence in daily life may require greater reliance on self-control to manage smartphone use and daily routines. The Interaction of Person-Affect-Cognition-Execution (I-PACE) model of specific Internet-use disorders proposes that vulnerability factors, including specific personality traits and motives for use, play an important role in the development and maintenance of addictive Internet-use behaviors (Brand et al., 2016). From this theoretical perspective, individuals with lower self-control may have greater difficulty resisting the urge to use smartphones. Previous studies have found a negative association between self-control and SA, with lower self-control being associated with more severe SA (He et al., 2025; Liu, 2023). Poor self-control has also been considered an important psychological factor associated with addictive behaviors (Stacy and Wiers, 2010). Individuals with poor self-control may find it more difficult to resist the attraction of smartphone content, functions, and games, making excessive smartphone use more likely (Wang Q. et al., 2024). In addition, self-control is closely related to sleep. Individuals with poor self-control may have greater difficulty resisting temptations, which may be associated with sleep problems and poorer sleep quality (Ge et al., 2021). A previous study has also reported a significant negative association between self-control and insomnia symptoms (Zhao et al., 2022). Notably, self-control may not only be separately associated with smartphone use and sleep but may also be involved in the relationship between them. A longitudinal study found that self-control played a mediating role in the relationship between problematic smartphone use and bedtime procrastination (Chen et al., 2023). These findings suggest complex relationships among self-control, smartphone use, and sleep problems.

Attentional bias refers to the differential allocation of attention to specific stimuli and is an aspect of selective attention (Stojek et al., 2018). Negative attentional bias is an important concept in cognitive psychology and refers to a tendency to preferentially perceive and allocate attention to negative rather than positive stimuli (Tian et al., 2025). Previous studies have shown that negative attentional bias is closely associated with depression, anxiety, and cognitive problems (Beevers et al., 2021; Li and Li, 2022). Stronger negative attentional bias may make individuals more likely to continuously focus on negative information and may be associated with higher levels of anxiety and depressive symptoms (Li and Li, 2022), which are in turn closely associated with poorer sleep (Zou et al., 2020). In addition to this potential indirect link, negative attentional bias itself is also associated with sleep. Previous research has found that greater negative attentional bias is significantly associated with poorer sleep quality (Yong et al., 2026). Meanwhile, negative attentional bias is also closely related to SA. A study of 891 college students found a significant positive association between negative attentional bias and SA, with depression mediating this relationship (Tian et al., 2025). These findings suggest that negative attentional bias may be associated with both SA and sleep problems. Taken together, self-control, SA, and negative attentional bias may be interrelated and reflect different aspects of college students’ behavioral regulation, smartphone use, and cognitive processing. These characteristics may have complex associations with their sleep.

Although previous studies have separately examined the relationships of self-control, SA, and negative attentional bias with sleep problems, these factors have typically been investigated in isolation, and their joint patterns at the individual level and their associations with insomnia symptoms remain unclear. Previous studies have begun to integrate Latent Profile Analysis (LPA) and network analysis to simultaneously examine individual heterogeneity in psychological and behavioral characteristics (Kabadayi, 2024; Peng and Liao, 2023). LPA, a person-centered approach, can identify latent subgroups characterized by different combinations of psychological and behavioral characteristics, thereby revealing individual heterogeneity that may be overlooked by traditional variable-centered approaches. However, LPA primarily focuses on patterns of individual characteristics and does not further reveal the structure of associations among psychological and behavioral factors within each latent profile. In contrast, network analysis can examine the complex associations among different factors and identify central and bridge nodes within the network. Therefore, this study combined LPA and network analysis to identify latent profiles of self-control, SA, and negative attentional bias among college students and further examine the network structure of self-control, SA, negative attentional bias, and insomnia symptoms in the overall sample and across the identified latent profiles. This integration provides both a person-centered perspective on individual differences and a network perspective on the interrelationships among the study variables.

Based on previous research and theoretical considerations, we hypothesized that (1) college students would exhibit different joint patterns of self-control, SA, and negative attentional bias, and (2) profiles characterized by lower self-control, higher SA, and higher negative attentional bias would be associated with more severe insomnia symptoms. Given the limited prior evidence regarding network structures across latent profiles, the network characteristics within each profile and potential variations across profiles were further explored.

## 2 Methods

### 2.1 Study participants

This cross-sectional online survey used convenience sampling to recruit participants from three universities in Wuhan, Hubei Province, China, between March and June 2024, including Hubei University of Chinese Medicine, Wuhan University of Science and Technology, and China University of Geosciences (Wuhan). The three universities were selected through convenience sampling based on accessibility and feasibility of data collection. An electronic questionnaire was distributed through the QuestionStar platform (a professional company that conducts online surveys). Participants were recruited primarily through general compulsory courses. During class breaks, course instructors briefly introduced the study and displayed a QR code linking to the online questionnaire. Students who were willing to participate voluntarily scanned the QR code and completed the questionnaire via QuestionStar. Before completing the questionnaire, participants read the study information and voluntarily participated after providing informed consent. The inclusion criteria were as follows: (1) being a full-time university student; (2) being able to independently use a smartphone to complete the electronic questionnaire; and (3) voluntarily agreeing to participate in the study. Participants were excluded if they self-reported a previous physician diagnosis of a severe mental or cognitive disorder.

One attention-check item was included in the questionnaire, which required participants to select option “A.” Questionnaires that did not meet this requirement were excluded. Questionnaires completed in less than 2 min or showing obvious straight-lining response patterns were also excluded. A total of 1,651 questionnaires were collected. Of these, 16 were completed in less than 2 min, as such a short duration was considered insufficient for careful completion of all questionnaire items, consistent with previous online survey studies that used a completion time of less than 2 min as a criterion for identifying potentially invalid responses (Guo et al., 2022; Yu et al., 2021); 14 showed straight-lining response patterns, defined as selecting the same response option consecutively across a substantial number of items despite the inclusion of both positively and negatively worded items; and 24 failed the attention check. After excluding 54 invalid questionnaires, 1,597 participants were included in the final analysis. To minimize duplicate participation, the online survey platform was configured to restrict multiple submissions from the same device.

### 2.2 Measurements

#### 2.2.1 Smartphone addiction scale for college students

The Smartphone Addiction Scale for College Students (SAS-C) was developed by Su et al. (2014) to assess SA among college students. The scale consists of 22 items across six dimensions: withdrawal, salience, social comfort, negative effects, app use, and app updates. Each item is rated on a 5-point Likert scale ranging from 1 (strongly disagree) to 5 (strongly agree), with a total score ranging from 22 to 110. Higher scores indicate a higher level of SA. In the original study, the Cronbach’s α coefficient was 0.88 for the total scale and ranged from 0.44 to 0.85 across the six dimensions. In the present study, the Cronbach’s α coefficient for the total scale was 0.947.

#### 2.2.2 Self-control scale

The Self-Control Scale (SCS) was developed by Tangney et al. (2004) and was revised by Tan and Guo (2008) for use among Chinese college students. It was used to assess self-control among college students. The scale consists of 19 items across five dimensions: impulse control, healthy habits, resisting temptation, concentration on work, and restraint of entertainment. Each item is rated on a 5-point Likert scale ranging from 1 (strongly disagree) to 5 (strongly agree), with a total score ranging from 19 to 95. Items 1, 5, 11, and 14 are positively scored, while the remaining items are reverse scored. Higher total scores indicate better self-control. In the present study, the Cronbach’s α coefficient of the scale was 0.797.

#### 2.2.3 Attention to positive and negative information scale

The Attention to Positive and Negative Information Scale (APNI) was developed by Noguchi et al. (2006) to assess the attentional bias of individuals toward negative or positive information. Lv et al. (2016) revised the scale for use in Chinese populations. The scale consists of 30 items across two dimensions: positive attentional bias (19 items) and negative attentional bias (11 items). Each item is rated on a 5-point Likert scale ranging from 1 (strongly disagree) to 5 (strongly agree). In the present study, only the negative attentional bias dimension (APNI-N) was used, which consists of 11 items, with a total score ranging from 11 to 55. Higher scores indicate a stronger attentional bias toward negative information. The Cronbach’s α coefficient for this dimension was 0.83. In the present study, the Cronbach’s α coefficient for APNI-N was 0.902.

#### 2.2.4 Athens insomnia scale

The Athens Insomnia Scale (AIS) contains eight items assessing sleep onset, nighttime and early morning awakenings, sleep quality, and the impact of insomnia on daily functioning (Soldatos et al., 2000). The AIS uses a 4-point scoring system, ranging from 0 (no problem) to 3 (severe problem), with a total score ranging from 0 to 24. The scale assesses participants’ subjective sleep quality during the past month. Higher scores indicate more severe insomnia symptoms, while lower scores indicate better sleep quality. The AIS has demonstrated good reliability and validity in Chinese populations, with a Cronbach’s α coefficient of 0.81 and a test–retest reliability coefficient of 0.80 (Chung et al., 2011). The Cronbach’s α coefficient of the scale was 0.830 in the present study.

### 2.3 Covariates

Covariates were selected based on previous studies on SA among college students and included gender, grade, major, place of residence, and only-child status (Tao et al., 2024; Xu and Li, 2026). Given the focus of the present study on insomnia symptoms, the number of dormitory residents and bedtime were additionally included as covariates. Gender was categorized as male or female; grade as freshman, sophomore, or junior and above; major as literature, science, engineering, agriculture, medicine, or other; place of residence as rural or urban; only-child status as yes or no; the number of dormitory residents as 1, 2, 3, 4, 5, or 6; and bedtime as before 22:00, 22:00–23:00, 23:00–24:00, or after 24:00. The results of the multicollinearity assessment are presented in Supplementary Table S1. All generalized variance inflation factor (GVIF) values were below 5, indicating no substantial multicollinearity in the model.

## 3 Statistical analysis

Latent profile analysis (LPA) was performed using Mplus version 8.0. The total scores of SA, SCS, and negative attentional bias (APNI-N) were used as observed indicators. Before the LPA, the three indicators were standardized into Z-scores. Models with one to five classes were then fitted to identify latent subgroups with different characteristics. Model fit was evaluated using the Akaike information criterion (AIC), Bayesian information criterion (BIC), sample-size adjusted Bayesian information criterion (aBIC), entropy, bootstrap likelihood ratio test (BLRT), and Lo–Mendell–Rubin adjusted likelihood ratio test (LMR-LRT). Lower AIC, BIC, and aBIC values indicated better model fit, while entropy values closer to 1 indicated better classification accuracy. The LMR-LRT and BLRT were used to compare a k-class model with a (k − 1)-class model, with a significant result indicating that the k-class model provided a better fit. The optimal model was determined by considering these indices together with class size, interpretability, and theoretical relevance. To reduce the risk of local maxima and improve the stability of model estimation, 500 random sets of starting values were used, with 100 retained for final-stage optimization. After the optimal profile solution was determined, participants were assigned to their most likely profile based on the highest posterior probability of profile membership. The resulting profile assignments were used in the subsequent regression and profile-specific network analyses. The final three-profile solution had an entropy of 0.848, and the average posterior probabilities for the three profiles were 0.944, 0.860, and 0.908, respectively, indicating generally good classification quality (Supplementary Table S2).

Except for LPA, all statistical analyses were performed using R software (version 4.5.3). After the optimal latent profile model was determined, baseline characteristics were compared across the latent profiles. Normally distributed continuous variables were presented as mean ± standard deviation (SD) and compared using one-way analysis of variance. Non-normally distributed continuous variables were presented as median and quartiles and compared using the Kruskal–Wallis test. Categorical variables were presented as frequencies and percentages [*n* (%)] and compared using the χ^2^ test. Multiple linear regression was then performed with the AIS total score as the dependent variable and latent profile membership as the independent variable. Class 3, which had the lowest AIS score, was used as the reference group. The associations between different latent profiles and insomnia symptoms were examined after adjustment for potential confounders. All questionnaire items were mandatory, and no item-level missing data were present in the analytic sample. All statistical tests were two-sided, and *p* < 0.05 was considered statistically significant.

Network analysis was conducted using the “bootnet” and “qgraph” packages in R to examine the associations among self-control, SA, negative attentional bias, and insomnia symptoms (Zhang et al., 2026). A total of 13 nodes were included in the network: 5 dimensions of the SCS (SCS1–SCS5), 6 dimensions of the SA scale (SA1–SA6), the negative attentional bias total score (APNI-N), and the AIS total score (AIS). The respective dimension scores of the SCS and SA were used as network nodes. As APNI-N and AIS were not further divided into separate dimensions, their total scores were used as network nodes. Based on the Gaussian graphical model (GGM), a regularized partial correlation network was estimated using the graphical least absolute shrinkage and selection operator (glasso) (Li et al., 2025). The Extended Bayesian Information Criterion (EBIC) was used to select the optimal network structure, with the EBIC tuning parameter set to 0.5. This method shrinks edge weights and reduces weaker edges to zero, resulting in a sparse and interpretable network (Epskamp et al., 2018). Nodes represented scale dimensions or composite scores, whereas edges represented regularized partial correlations between pairs of nodes after controlling for all other nodes in the network (Epskamp et al., 2018). Edge thickness indicated the strength of the association, while edge color indicated the direction of the association.

Strength centrality, betweenness centrality, closeness centrality, and expected influence (EI) were calculated to assess node centrality. EI was mainly used to identify central nodes in the network. EI is the sum of all edge weights between a node and its neighboring nodes while retaining the positive and negative signs of the edge weights. A higher EI value indicates a greater overall influence of the node in the network (Robinaugh et al., 2016). The “networktools” package was further used to divide the nodes into four communities: SCS, SA, APNI-N, and AIS. Bridge strength was calculated to identify key bridge nodes connecting different communities. Bridge strength reflects the overall strength of a node’s connections with nodes in other communities (Jones et al., 2021). Higher bridge strength values indicate stronger connections between a node and other communities.

The “bootnet” package in R was used to assess the accuracy of network edge weights and the stability of centrality indices (Epskamp et al., 2018). A non-parametric bootstrap with 1,000 samples was used to estimate the 95% confidence intervals (95% CIs) of the edge weights, with narrower confidence intervals indicating more accurate estimates. A case-dropping bootstrap with 1,000 samples was used to assess the stability of centrality indices and to calculate the correlation stability (CS) coefficient. The CS coefficient represents the maximum proportion of the sample that can be dropped while maintaining a correlation of at least 0.70 between the centrality estimates and those of the original network. A CS coefficient ≥0.25 is generally considered acceptable, while a value ≥0.50 indicates good stability (Wang D. et al., 2024). In addition, bootstrap difference tests were used to compare EI and bridge strength across nodes.

In addition, to further explore network characteristics across different latent profiles, participants were stratified according to the profiles identified by LPA, and separate network models were constructed for each profile. These networks were used to examine the associations among SCS, SA, APNI-N, and AIS, as well as key node characteristics within each profile. The Network Comparison Test (NCT) was also used to compare the networks of different latent profiles pairwise and to examine differences in network structure and global strength (Lin et al., 2026). *p* values from pairwise network comparisons were adjusted using the Benjamini–Hochberg false discovery rate procedure.

## 4 Results

### 4.1 Common method bias

Harman’s single-factor test was conducted to assess common method bias. All measurement items were included in an unrotated exploratory factor analysis. The first factor explained 31.10% of the total variance, which was below the recommended threshold of 40%, indicating that common method bias was not a serious concern in this study.

### 4.2 Identification and characteristics of the latent profiles

Models with one to five classes were fitted sequentially (Table 1). As the number of classes increased, the AIC, BIC, and aBIC values continued to decrease, and the LMR and BLRT were significant for the two- to five-class models (all *p* < 0.001). The three-class model had an entropy value of 0.848, which was higher than those of the four-class (0.827) and five-class (0.822) models, indicating better classification accuracy. Further comparison of the three- and four-class solutions showed that, although the four-class solution had lower information criteria, its classification accuracy did not improve, and the additional class mainly represented a further subdivision along the shared gradient of self-control, SA, and negative attentional bias, rather than a clearly distinct pattern of characteristics. Thus, the additional model complexity provided limited additional interpretive value. In the five-class model, the smallest class accounted for only 2.40% of the total sample, indicating a relatively small class size. The diagonal average posterior probabilities for the three classes were 0.944, 0.860, and 0.908, respectively, all exceeding the conventional threshold of 0.70 (Li et al., 2026). The complete posterior probability matrix is presented in Supplementary Table S2. Considering model fit, classification accuracy, class size, and interpretability, the three-class model was selected as the optimal model.

**Table 1:** Fit indices for latent profile models with different numbers of profiles.

class	AIC	BIC	aBIC	Entropy	LMR (*P*)	BLRT (*P*)	Proportion (%)
1	13608.269	13640.524	13621.464	—		—	—
2	12627.764	12681.523	12649.755	0.799	<0.001	<0.001	20.66/79.34
3	12010.817	12086.080	12041.604	0.848	<0.001	<0.001	75.20/10.83/13.96
4	11719.698	11816.464	11759.281	0.827	<0.001	<0.001	64.68/5.26/7.01/23.04
5	11542.952	11661.221	11591.332	0.822	<0.001	<0.001	59.05/19.72/13.53/5.30/2.40

AIC, Akaike information criterion; BIC, Bayesian information criterion; aBIC, sample-size adjusted Bayesian information criterion; BLRT, bootstrap likelihood ratio test; LMR, Lo–Mendell–Rubin adjusted likelihood ratio test; Entropy, classification accuracy; Proportion, percentage of participants in each latent profile.

As shown in Figure 1 and Supplementary Table S3, Class 1 included 1,201 participants (75.20%) and showed relatively moderate levels of self-control, SA, and negative attentional bias. This class was therefore named the “Moderate Self-Control–Moderate SA and Negative Attention Profile.” Class 2 included 173 participants (10.83%) and had the lowest self-control score (44.3) and the highest SA (87.8) and negative attentional bias (45.5) scores. This class was named the “Low Self-Control–High SA and Negative Attention Profile.” Class 3 included 223 participants (13.96%) and had the highest self-control score (73.3) and the lowest SA (34.2) and negative attentional bias (23.1) scores. This class was named the “High Self-Control–Low SA and Negative Attention Profile.”

**Figure 1:**
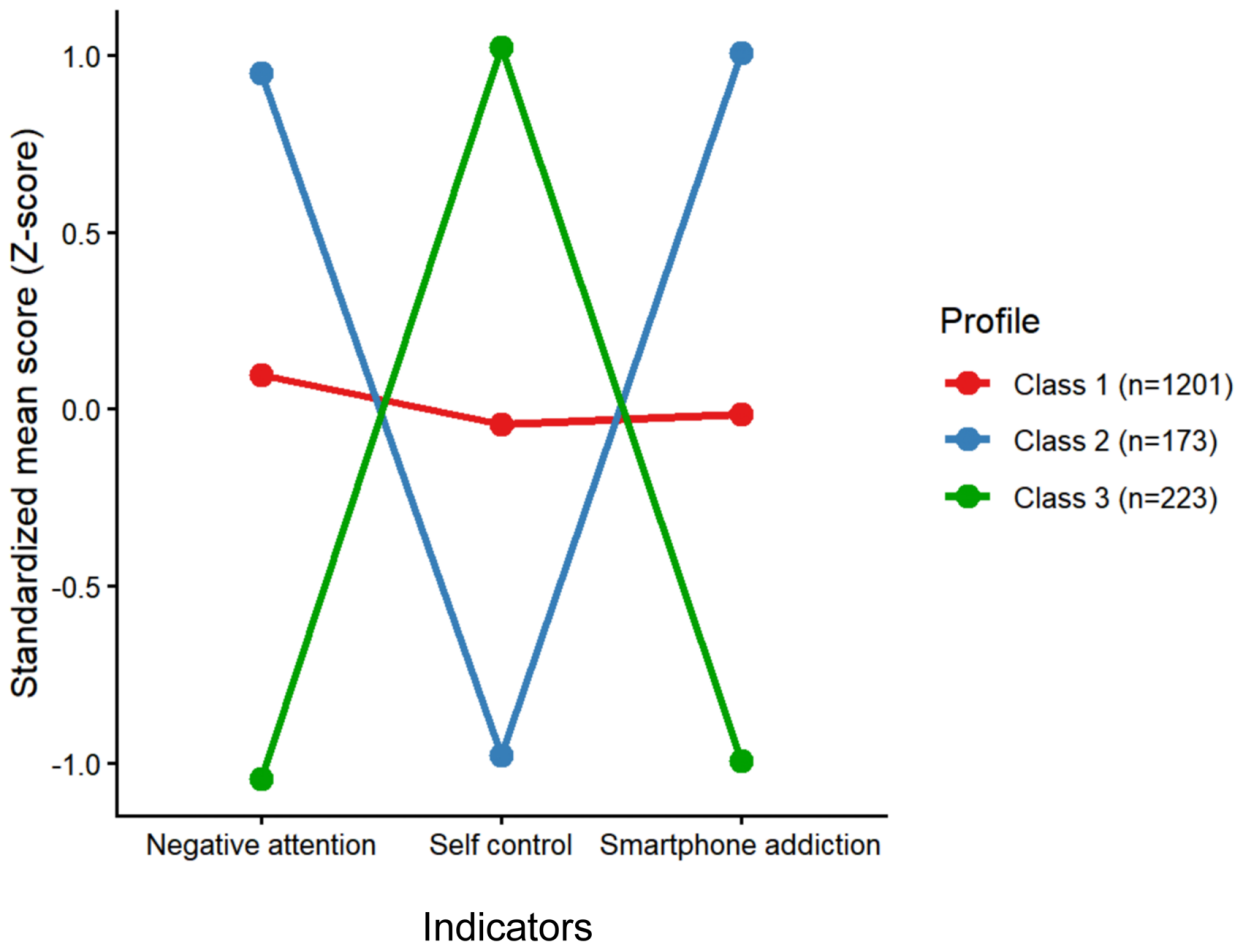
Standardized mean profiles of self-control, smartphone addiction, and negative attention across three latent profiles. Class 1: “Moderate Self-Control–Moderate SA and Negative Attention Profile”; Class 2: “Low Self-Control–High SA and Negative Attention Profile”; Class 3: “High Self-Control–Low SA and Negative Attention Profile”.

### 4.3 Baseline characteristics

A total of 1,597 college students were included in the study, of whom 1,098 (68.8%) were female, 903 (56.5%) were from rural areas, 1,029 (64.4%) were freshmen, 560 (35.1%) were sophomores, and 8 (0.5%) were juniors or above. In addition, 55.9% were medical students, and 658 (41.2%) usually went to bed after 24:00. Significant differences were observed across the latent profiles in place of residence, grade, major, bedtime, and number of dormitory residents (all *p* < 0.05). AIS scores also differed significantly across the latent profiles (*p* < 0.001). The Low Self-Control–High SA and Negative Attention Profile had the highest AIS score (7.99 ± 5.13), followed by the Moderate Self-Control–Moderate SA and Negative Attention Profile (6.04 ± 3.73), while the High Self-Control–Low SA and Negative Attention Profile had the lowest score (3.56 ± 3.17) (Table 2).

**Table 2:** Characteristics of participants according to latent profiles.

Characteristic	Overall (*N* = 1,597)	Class			*p* value
		1 (*N* = 1,201)	2 (*N* = 173)	3 (*N* = 223)	
Gender					0.058
Male	499 (31.2%)	362 (30.1%)	52 (30.1%)	85 (38.1%)	
Female	1,098 (68.8%)	839 (69.9%)	121 (69.9%)	138 (61.9%)	
Place of residence					0.001
Urban	694 (43.5%)	493 (41.0%)	79 (45.7%)	122 (54.7%)	
Rural	903 (56.5%)	708 (59.0%)	94 (54.3%)	101 (45.3%)	
Grade level					0.002
Freshman	1,029 (64.4%)	788 (65.6%)	88 (50.9%)	153 (68.6%)	
Sophomore	560 (35.1%)	408 (34.0%)	84 (48.6%)	68 (30.5%)	
Junior or above	8 (0.5%)	5 (0.4%)	1 (0.6%)	2 (0.9%)	
Major					0.013
Literature	218 (13.7%)	157 (13.1%)	30 (17.3%)	31 (13.9%)	
Science	327 (20.5%)	237 (19.7%)	32 (18.5%)	58 (26.0%)	
Engineering	62 (3.9%)	41 (3.4%)	15 (8.7%)	6 (2.7%)	
Agriculture	4 (0.3%)	3 (0.2%)	1 (0.6%)	0 (0.0%)	
Medicine	892 (55.9%)	687 (57.2%)	88 (50.9%)	117 (52.5%)	
Others	94 (5.9%)	76 (6.3%)	7 (4.0%)	11 (4.9%)	
Only child					0.904
Yes	534 (33.4%)	398 (33.1%)	59 (34.1%)	77 (34.5%)	
No	1,063 (66.6%)	803 (66.9%)	114 (65.9%)	146 (65.5%)	
Bedtime					<0.001
Before 22:00	34 (2.1%)	14 (1.2%)	8 (4.6%)	12 (5.4%)	
22:00–23:00	190 (11.9%)	139 (11.6%)	14 (8.1%)	37 (16.6%)	
23:00–24:00	715 (44.8%)	554 (46.1%)	53 (30.6%)	108 (48.4%)	
After 24:00	658 (41.2%)	494 (41.1%)	98 (56.6%)	66 (29.6%)	
Number of dormitory residents					<0.001
1	11 (0.7%)	2 (0.2%)	5 (2.9%)	4 (1.8%)	
2	6 (0.4%)	6 (0.5%)	0 (0.0%)	0 (0.0%)	
3	57 (3.6%)	42 (3.5%)	6 (3.5%)	9 (4.0%)	
4	696 (43.6%)	531 (44.2%)	62 (35.8%)	103 (46.2%)	
5	87 (5.4%)	68 (5.7%)	4 (2.3%)	15 (6.7%)	
6	740 (46.3%)	552 (46.0%)	96 (55.5%)	92 (41.3%)	
Self-control score	58.54 ± 9.93	57.85 ± 6.77	44.31 ± 6.35	73.28 ± 7.16	<0.001
Smartphone addiction score	59.77 ± 17.09	60.47 ± 10.63	87.82 ± 12.66	34.23 ± 9.77	<0.001
Negative attention score	35.18 ± 8.38	35.94 ± 5.78	45.52 ± 5.90	23.05 ± 7.95	<0.001
Athens insomnia scale score	5.90 ± 4.00	6.04 ± 3.73	7.99 ± 5.13	3.56 ± 3.17	<0.001

Data are presented as *n* (%) or mean ± standard deviation (SD). *p* values were calculated using chi-square test for categorical variables and one-way analysis of variance (ANOVA) for continuous variables; Class 1: “Moderate Self-Control–Moderate SA and Negative Attention Profile”; Class 2: “Low Self-Control–High SA and Negative Attention Profile”; Class 3: “High Self-Control–Low SA and Negative Attention Profile”.

### 4.4 Associations between latent profiles and AIS scores

Multicollinearity diagnostics showed that the adjusted GVIF values ranged from 1.02 to 1.10, indicating no obvious multicollinearity in the regression model (Supplementary Table S1). Multiple linear regression analysis showed that, after adjustment for relevant covariates, the Moderate Self-Control–Moderate SA and Negative Attention Profile was significantly associated with a higher AIS score compared with the High Self-Control–Low SA and Negative Attention Profile (*B* = 2.31, 95% CI: 1.75–2.86, *p* < 0.001). Similarly, the Low Self-Control–High SA and Negative Attention Profile was significantly associated with a higher AIS score (*B* = 4.04, 95% CI: 3.27–4.81, *p* < 0.001) (Table 3).

**Table 3:** Multivariable linear regression analysis of the associations between latent profile membership and athens insomnia scale scores.

Latent profile	AIS score	
	*B* (95% CI)	*p*
Class 1	2.31 (1.75, 2.86)	<0.001
Class 2	4.04 (3.27, 4.81)	<0.001
Class 3	Reference	—

Class 1: “Moderate Self-Control–Moderate SA and Negative Attention Profile”; Class 2: “Low Self-Control–High SA and Negative Attention Profile”; Class 3: “High Self-Control–Low SA and Negative Attention Profile”; *B*, unstandardized regression coefficient; CI, confidence interval; AIS, Athens Insomnia Scale. Class 3 was used as the reference group. The model was adjusted for gender, grade, major, place of residence, only-child status, number of dormitory residents and bedtime.

### 4.5 Network analysis

#### 4.5.1 Network structure and visualization

In the overall sample network, 13 nodes and 78 possible edges were included, of which 66 had non-zero weights (Figure 2). The network density was 0.846, and the mean edge weight was 0.091. In the profile-specific networks (Supplementary Figure S1), the Moderate Self-Control–Moderate SA and Negative Attention Profile (Class 1) contained 51 non-zero edges, with a network density of 0.654 (51/78) and a mean edge weight of 0.085. The Low Self-Control–High SA and Negative Attention Profile (Class 2) contained 41 non-zero edges, with a network density of 0.526 (41/78) and a mean edge weight of 0.120. The High Self-Control–Low SA and Negative Attention Profile (Class 3) contained 35 non-zero edges, with a network density of 0.449 (35/78) and a mean edge weight of 0.101.

**Figure 2:**
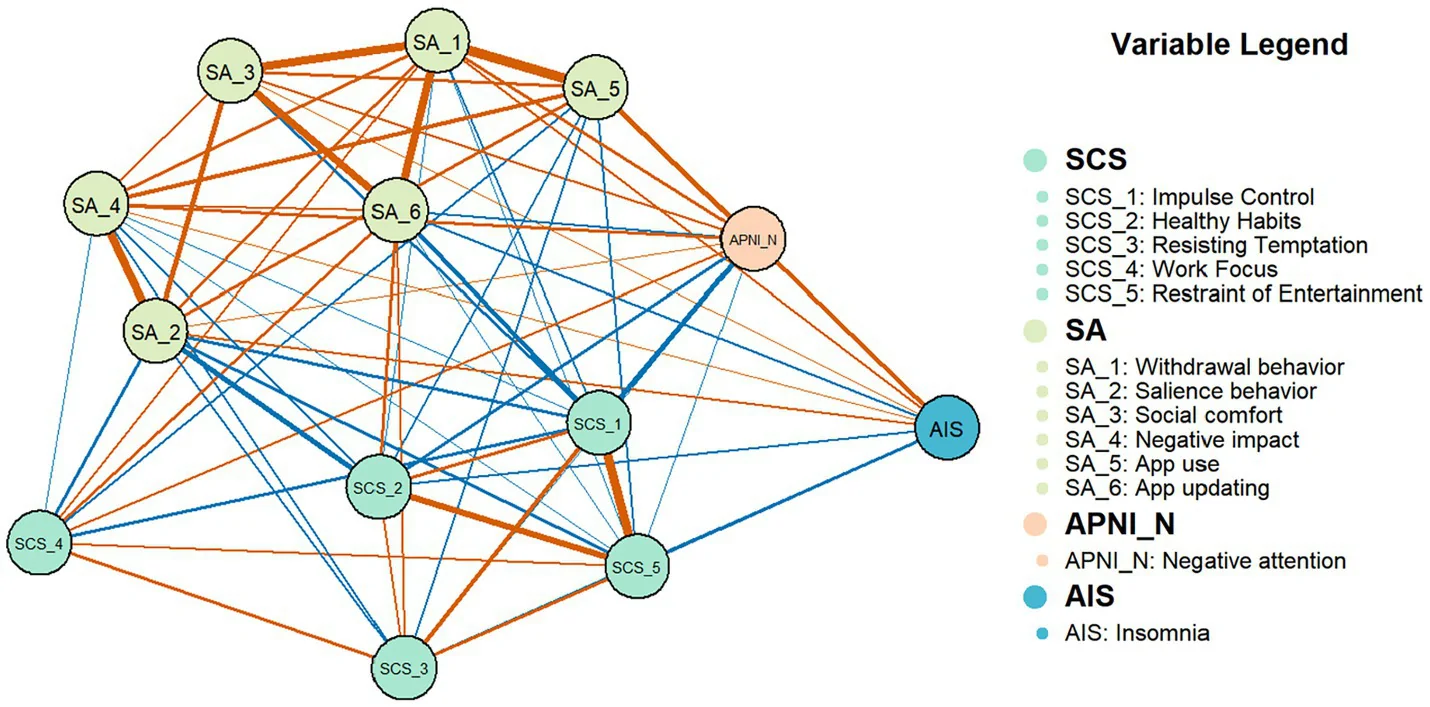
Network structure of self-control, smartphone addiction, negative attention, and insomnia symptoms among college students. Orange edges indicate positive associations, whereas blue edges indicate negative associations; SCS, self-control scale; SA, smartphone addiction; APNI_N, negative attention; AIS, Athens Insomnia Scale.

#### 4.5.2 Edge weights

In the overall sample network, the strongest within-community connection in the SA community was observed between SA1 (Withdrawal behavior) and SA5 (App use) (weight = 0.397). In the self-control community, the strongest connection was between SCS1 (Impulse Control) and SCS5 (Restraint of Entertainment) (weight = 0.327). Among the cross-community connections, the strongest negative connection was observed between APNI-N (Negative attention) and SCS1 (Impulse Control) (weight = −0.161), followed by the negative connection between SA2 (Salience behavior) and SCS2 (Healthy Habits) (weight = −0.158). For AIS, the strongest cross-community connection was observed with APNI-N (Negative attention), and this association was positive (weight = 0.117).

The profile-specific networks showed both similarities and differences in their connection patterns. In the Moderate Self-Control–Moderate SA and Negative Attention Profile (Class 1), the strongest connection was observed between SA1 (Withdrawal behavior) and SA5 (App use) (weight = 0.385). Among the cross-community connections, the strongest negative connection was between SA2 (Salience behavior) and SCS2 (Healthy Habits) (weight = −0.145), followed by the positive connection between AIS (Insomnia) and APNI-N (Negative attention) (weight = 0.115). In the Low Self-Control–High SA and Negative Attention Profile (Class 2), the strongest connection was observed between SA1 (Withdrawal behavior) and SA6 (App updating) (weight = 0.395). The strongest cross-community connection was the negative connection between SA2 (Salience behavior) and SCS2 (Healthy Habits) (weight = −0.149), followed by the negative connection between SA4 (Negative impact) and SCS5 (Restraint of Entertainment) (weight = −0.145). In the High Self-Control–Low SA and Negative Attention Profile (Class 3), the strongest connection was observed between SA1 (Withdrawal behavior) and SA5 (App use) (weight = 0.366). Among the cross-community connections, the strongest was the positive connection between APNI-N (Negative attention) and SA5 (App use) (weight = 0.113), followed by the negative connection between SA2 (Salience behavior) and SCS2 (Healthy Habits) (weight = −0.087).

#### 4.5.3 Centrality and bridge centrality indices

EI was used as the primary measure of node centrality. In the overall sample network, SA1 (Withdrawal behavior, EI = 1.263) had the highest EI, followed by SA3 (Social comfort, EI = 0.795) and SA5 (App use, EI = 0.679), indicating that SA1 had the highest centrality in the overall network (Figure 3). The centrality difference results (Supplementary Figure S2) indicated that these nodes were statistically stronger than the others.

**Figure 3:**
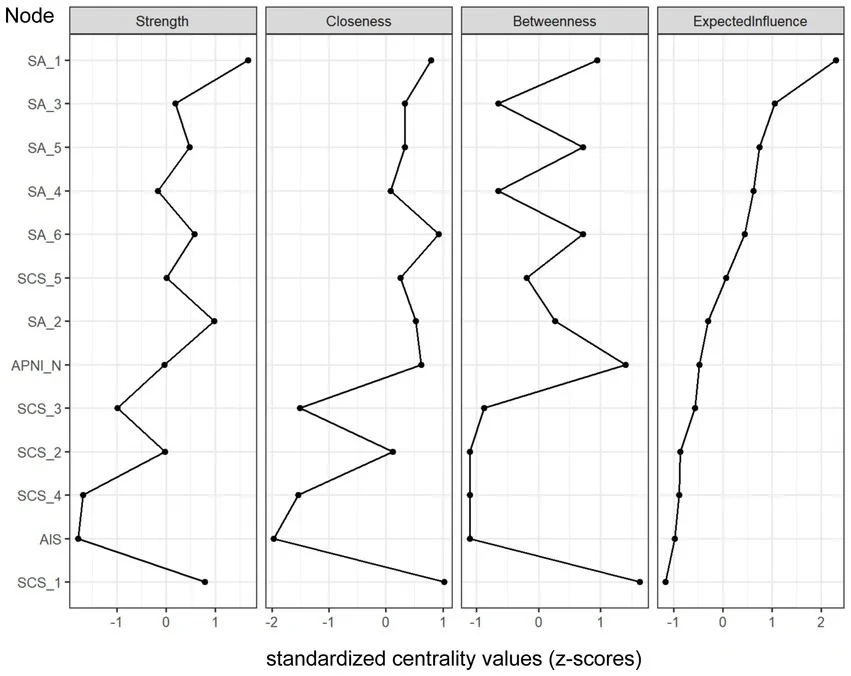
Standardized centrality indices of the network of self-control, smartphone addiction, negative attention, and insomnia symptoms among college students. Centrality indices are presented as z-scores, with higher values indicating greater centrality within the estimated network. Expected influence was treated as the primary signed centrality index because the network contained both positive and negative edges. Strength, closeness, and betweenness are presented for descriptive comparison; SCS, self-control scale; SA, smartphone addiction; APNI_N, negative attention; AIS, Athens Insomnia Scale.

The centrality patterns of the three latent profiles are shown in Supplementary Figure S3. In the Moderate Self-Control–Moderate SA and Negative Attention Profile (Class 1), SA1 (Withdrawal behavior, EI = 1.130) had the highest EI, followed by SA3 (Social comfort, EI = 0.710) and SA5 (App use, EI = 0.626). In the Low Self-Control–High SA and Negative Attention Profile (Class 2), SA1 (Withdrawal behavior, EI = 1.169) had the highest EI, followed by SA6 (App updating, EI = 0.863) and SA3 (Social comfort, EI = 0.853). In the High Self-Control–Low SA and Negative Attention Profile (Class 3), SA1 (Withdrawal behavior, EI = 1.026) had the highest EI, followed by SA5 (App use, EI = 0.689) and SA4 (Negative impact, EI = 0.658). Although the other nodes with high EI values varied across the three profiles, SA1 (Withdrawal behavior) consistently had the highest EI in all three profiles.

The bridge centrality results are shown in Figure 4. SA1 (Withdrawal behavior, bridge strength = 1.294) had the highest bridge strength, followed by APNI-N (Negative attention, bridge strength = 0.915), indicating that these two nodes had stronger connections with nodes in other communities and may serve as key bridge nodes in the network. Bootstrap difference tests showed significant differences in bridge strength between some nodes (Supplementary Figure S4).

**Figure 4:**
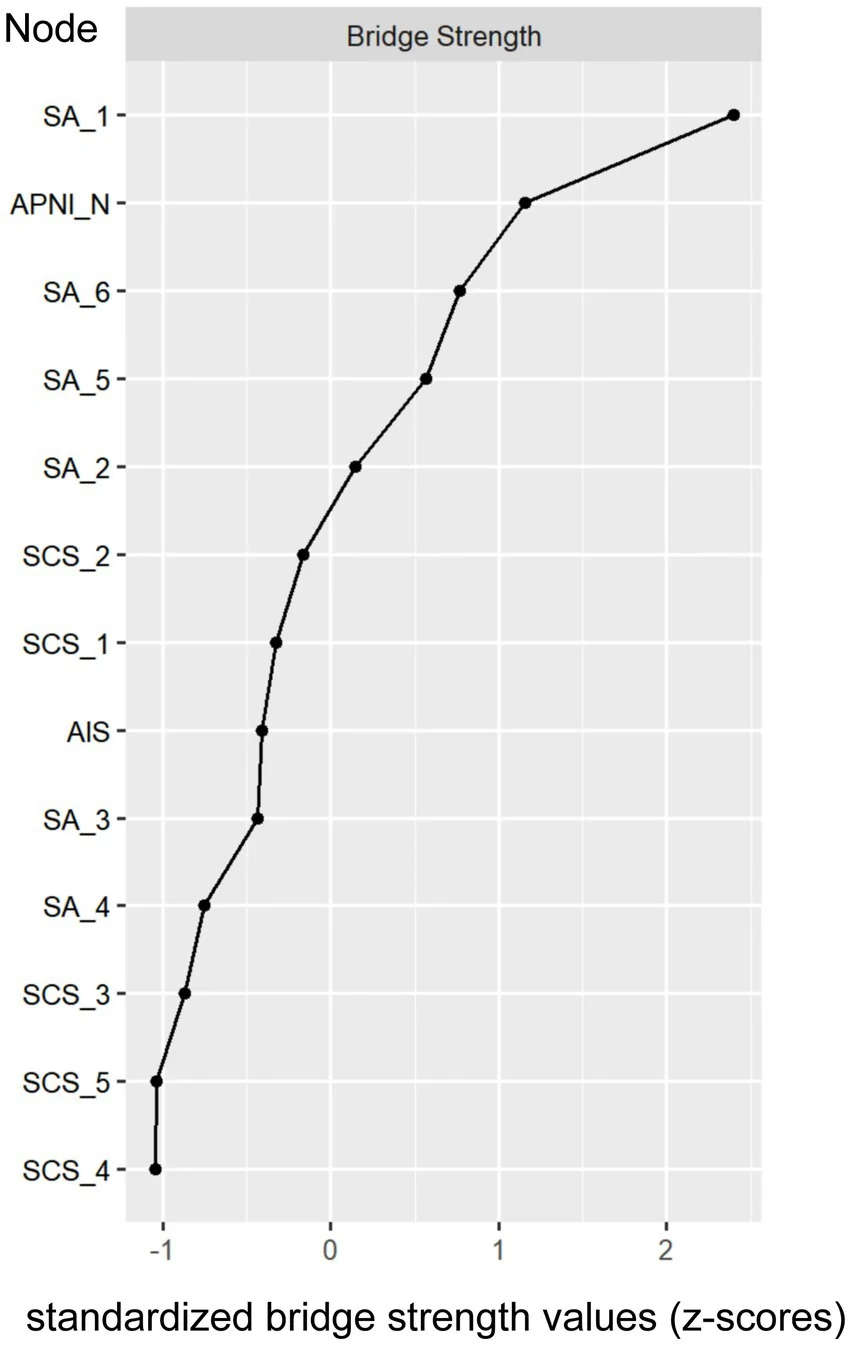
Bridge centrality of the network of self-control, smartphone addiction, negative attention, and insomnia symptoms among college students. Bridge strength values are presented as z-scores. SCS, self-control scale; SA, smartphone addiction; APNI_N, negative attention; AIS, Athens Insomnia Scale.

#### 4.5.4 Network stability

Supplementary Figure S5 shows the accuracy of edge-weight estimates assessed using a nonparametric bootstrap analysis. The 95% CIs for most edge weights were relatively narrow, indicating good accuracy of the edge-weight estimates. Supplementary Figure S6 shows the results of the centrality stability analysis using a case-dropping bootstrap. In the overall sample network, the CS coefficients for both EI and bridge strength were 0.75, indicating good stability of both centrality measures. Across the profile-specific networks, the CS coefficients for EI were 0.75, 0.44, and 0.52 for Class 1, Class 2, and Class 3, respectively, all meeting the acceptable stability criterion. The corresponding CS coefficients for bridge strength were 0.13, 0.05, and 0.05, respectively, all below 0.25, indicating insufficient stability of bridge strength in the profile-specific networks. Therefore, the bridge centrality results for the profile-specific networks were not further interpreted.

#### 4.5.5 Network comparison across profiles

The NCT results showed no significant differences in network structure across the pairwise comparisons of the three profiles (Supplementary Table S4). Specifically, no significant differences were found between Class 1 and Class 2 (*M* = 0.199, unadjusted *p* = 0.173), between Class 1 and Class 3 (*M* = 0.156, unadjusted *p* = 0.468), or between Class 2 and Class 3 (*M* = 0.206, unadjusted *p* = 0.603). For global strength, the unadjusted comparison between Class 2 and Class 3 was statistically significant (4.91 vs. 3.53; *S* = 1.376, unadjusted *p* = 0.038), but this difference was no longer significant after false discovery rate (FDR) correction (adjusted *p* = 0.114). No significant differences in global strength were observed between Class 1 and Class 2 (*S* = 0.567, unadjusted *p* = 0.638) or between Class 1 and Class 3 (*S* = 0.809, unadjusted *p* = 0.176). After FDR correction, none of the pairwise differences in network structure or global strength were statistically significant.

## 5 Discussion

This study combined LPA and network analysis to examine the relationships among self-control, SA, negative attentional bias, and insomnia symptoms in college students. Three latent profiles were identified: the Moderate Self-Control–Moderate SA and Negative Attention Profile, the Low Self-Control–High SA and Negative Attention Profile, and the High Self-Control–Low SA and Negative Attention Profile. Compared with the High Self-Control–Low SA and Negative Attention Profile, the other two profiles were significantly associated with higher AIS scores. Network analysis identified SA1 (Withdrawal behavior) as the node with the highest EI in the overall network and across all three latent profiles, and it also exhibited the highest bridge strength in the overall network. APNI-N (Negative attention) showed relatively high bridge strength. Although some network characteristics varied across the latent profiles, the NCT showed no significant differences in overall network structure or global strength after FDR correction.

First, the three latent profiles showed a relatively clear gradient pattern, with lower self-control co-occurring with higher levels of SA and negative attentional bias, and higher self-control co-occurring with lower levels of these characteristics. This pattern suggests that these psychological and behavioral characteristics may cluster among college students. From the theoretical perspective of the I-PACE model, individual characteristics and affective, cognitive, and executive functions may jointly contribute to the development and maintenance of problematic Internet-use behaviors (Brand et al., 2016). Individuals with lower self-control may have greater difficulty inhibiting the immediate impulse to use smartphones and may be more inclined to pursue immediate gratification, current desires, and novel experiences (Wang Q. et al., 2024). Meanwhile, stronger negative attentional bias may make individuals more likely to attend to and process negative information, and may be accompanied by more negative emotional experiences (De Houwer and Koster, 2023). In this context, given their portability and immediate accessibility, smartphones may serve as a means of diverting attention or regulating negative emotions (Jiang and Zhao, 2016), which may co-occur with higher levels of SA. This is also consistent with previous findings on the associations of self-control and negative attentional bias with SA (Tian et al., 2025; Wang Q. et al., 2024). In contrast, individuals with higher self-control tend to voluntarily choose activities that provide long-term benefits and derive satisfaction from engaging in or completing these activities (Jiang and Zhao, 2016), and therefore may have a greater capacity to regulate smartphone use and attention. Overall, these findings suggest that self-control, SA, and negative attentional bias may not exist in isolation but rather show different joint patterns, primarily reflecting quantitative differences in their combined levels among college students. These findings support the first hypothesis.

The gradient pattern observed across the latent profiles was also reflected in insomnia symptoms, suggesting that the co-occurrence of lower self-control, higher SA, and stronger negative attentional bias may be associated with greater sleep difficulties. This finding is generally consistent with previous research on self-control, SA, negative attentional bias, and sleep (Ling et al., 2024; Liu et al., 2022; Yong et al., 2026) and further suggests that insomnia symptoms among college students may be associated with the co-occurrence of multiple psychological and behavioral characteristics. Lower self-control may make it more difficult for college students to maintain regular and healthy sleep habits and resist the urge to use smartphones before bedtime, thereby making them more prone to behaviors such as bedtime procrastination (Ling et al., 2024). Meanwhile, higher levels of SA may make it difficult for individuals to voluntarily stop or control smartphone use. Particularly when faced with heavy academic demands during the day, some college students may extend their nighttime smartphone use to meet their needs for entertainment or relaxation. Continued smartphone use before bedtime may delay bedtime and sleep onset and shorten actual sleep duration, while nighttime exposure to screen light may further disrupt normal sleep by suppressing melatonin secretion and affecting circadian rhythms (Yin et al., 2024; Zhang et al., 2023). In addition, stronger negative attentional bias may make individuals more likely to continuously attend to and process negative information and may be accompanied by more negative emotional experiences, all of which are closely associated with insomnia symptoms (Li and Li, 2022; Yong et al., 2026). Yong et al. (2026) further proposed that attentional bias toward threatening information may be accompanied by heightened cognitive arousal, thereby adversely affecting normal sleep. These findings support the second hypothesis and suggest that considering self-control, SA, and negative attentional bias together may contribute to a more comprehensive understanding of insomnia symptoms among college students.

Network analysis further revealed the associations between different psychological and behavioral characteristics and insomnia symptoms. A particularly consistent feature of the network analysis was the central position of SA1 (Withdrawal behavior) across the overall network and all three latent profiles. Withdrawal behavior primarily reflects discomfort and a strong urge to use a smartphone when access is restricted and represents an important feature of psychological dependence on smartphones (Su et al., 2014). This finding is consistent with previous network analysis studies. Tateno et al. (2022) found that withdrawal symptoms had the highest strength among college students, while another network study of SA among adolescents similarly identified withdrawal as having the highest EI (Liu et al., 2025). This finding may be understood from the perspective of negative reinforcement. When individuals stop using their smartphones, they may experience negative emotions or withdrawal-like experiences, whereas resuming smartphone use may temporarily alleviate such discomfort. This negative reinforcement process may contribute to the maintenance of smartphone use (Contractor et al., 2017). Empirical evidence has also shown that withdrawal-like experiences resulting from smartphone restriction may be accompanied by increased anxiety, particularly among individuals with frequent smartphone use or higher levels of smartphone dependence (Cheever et al., 2014). From the perspective of the I-PACE model, repeated craving, reinforcement learning, and reduced inhibitory control may further contribute to the persistence and habitualization of addictive behaviors (Brand et al., 2019). In addition, strong connections between SA1 (Withdrawal behavior) and SA5 (App use) were observed in the overall network and in Class 1 and Class 3, whereas the strongest connection in Class 2 was observed between SA1 and SA6 (App updating), suggesting that the local connections between withdrawal behavior and other SA characteristics may differ across latent profiles. Given that the NCT showed no significant differences in the overall network structure across profiles, these local differences should be interpreted with caution. Notably, SA1 also exhibited the highest bridge strength in the overall network, suggesting strong conditional connections with nodes from other communities. Given its consistently central position across the different latent profiles, withdrawal behavior may represent an important feature for understanding the complex associations of SA with self-control, negative attentional bias, and insomnia symptoms. However, centrality indices in the present cross-sectional network reflect the position of nodes within the estimated structure of conditional associations and do not indicate temporal direction or causal influence. Therefore, the high EI of withdrawal behavior should not be interpreted as evidence that it causally influences other nodes or necessarily represents a priority target for intervention.

In addition to SA1, APNI-N (Negative attention) exhibited the second-highest bridge strength in the overall network. Notably, the connection between AIS and APNI-N was the strongest cross-community connection involving AIS, consistent with the close association between negative attentional bias and insomnia symptoms (Yong et al., 2026). Meanwhile, APNI-N and SCS1 (Impulse Control) showed the strongest negative cross-community connection in the overall network, indicating a strong conditional association between higher negative attentional bias and lower impulse control. These findings suggest that negative attentional bias may be closely related not only to insomnia symptoms but also to self-control characteristics. However, the relatively high bridge strength of APNI-N should be interpreted cautiously. Because APNI-N was represented by a single total-score node, it constituted a singleton community. As a result, all of its connections with nodes from other communities were treated as between-community connections. This feature may have contributed to its relatively high bridge strength and may limit direct comparison with nodes belonging to multi-node communities. Thus, the bridge centrality of APNI-N should not be interpreted as evidence that negative attentional bias plays a uniquely important bridging role in the network.

Although the profile-specific networks showed some descriptive variation, the FDR-corrected NCT did not provide evidence of differences in overall network structure or global strength across the three latent profiles. One possible explanation is that LPA distinguishes subgroups primarily based on the levels and combined patterns of self-control, SA, and negative attentional bias, whereas network analysis focuses on conditional associations among variables. Therefore, marked differences in variable levels do not necessarily correspond to overall differences in their patterns of interconnections. Together with the clear gradient pattern observed across the three latent profiles, these findings do not provide sufficient evidence that the profiles represent qualitatively different psychological configurations. Instead, they may primarily reflect quantitative differences in the joint levels of self-control, SA, and negative attentional bias. In addition, the relatively small sample sizes of Classes 2 and 3 and the unequal sample sizes across profiles may have affected the precision of the profile-specific network estimates and reduced the statistical power of the NCT to detect between-profile differences. Therefore, these variations should be interpreted as descriptive rather than as evidence of differences in network structure across profiles.

This study has several strengths. First, we combined LPA and network analysis to examine the relationships among self-control, SA, negative attentional bias, and insomnia symptoms in college students. LPA enabled the identification of latent subgroups characterized by different combinations of psychological and behavioral characteristics, while network analysis further revealed conditional associations among these characteristics and identified nodes with high centrality and bridge centrality. Thus, combining these two approaches provides a more comprehensive understanding of the complex relationships among the four constructs at both the subgroup and network levels. Second, in addition to constructing the overall network, we further compared network characteristics across different latent profiles, providing additional insights into network patterns among students with different psychological and behavioral characteristics. Finally, the relatively large sample size and the good stability of EI and bridge strength in the overall network enhance the reliability of the main network findings.

This study also has several limitations. First, the cross-sectional design precludes causal inferences, and future longitudinal studies are needed to further examine the dynamic relationships among self-control, SA, negative attentional bias, and insomnia symptoms. Second, all variables were assessed using self-report questionnaires and may therefore be subject to recall bias and social desirability bias. Future studies could incorporate objective measures, such as smartphone screen time recorded by the device and wearable device–based sleep measures, to further validate the present findings. Third, the sample sizes differed substantially across the latent profiles, with relatively small samples in Classes 2 and 3. Although the EI estimates showed acceptable stability across all three profiles, the CS coefficients for bridge strength were below 0.25; therefore, profile-specific bridge centrality results were not further interpreted. The relatively small and unequal profile sizes may also have affected network estimation and the statistical power of the NCT to detect between-profile differences. Future studies with larger and more balanced samples across profiles are needed to replicate and validate the profile-specific network findings. Fourth, no formal clinical assessment of psychiatric or psychological conditions was conducted, and symptoms of depression and anxiety were not assessed as potential covariates. As these factors are closely related to sleep disturbances and problematic smartphone use, their omission may have resulted in residual confounding. Thus, the observed associations should be interpreted cautiously. Future studies should include validated measures of depression and anxiety and, where feasible, clinical assessments. Other potentially relevant factors, such as academic stress and physical activity, should also be considered to further reduce residual confounding. Fifth, this study used convenience sampling and recruited participants from only three universities in Wuhan, which may limit the representativeness and generalizability of the findings. Classroom-based recruitment may also have underrepresented students who were absent from class, potentially introducing selection bias. Moreover, because class-level identifiers were not collected, potential clustering of participants within classes could not be formally assessed or accounted for in the analyses. Given that students across different universities and regions may differ in smartphone use, self-control, attentional characteristics, and sleep, caution is needed when generalizing the identified latent profiles and observed associations to the broader college student population. Future studies involving universities from different regions and using stratified or probability-based sampling are warranted. Sixth, although the entropy and average posterior probabilities indicated generally good classification quality, subsequent analyses were based on most-likely profile assignment and therefore did not explicitly account for classification uncertainty. Potential misclassification may therefore have affected the estimated associations in the regression analyses and the composition and parameter estimates of the profile-specific networks. In addition, exact age was not collected in this study, and possible age differences across the latent profiles could therefore not be examined.

## 6 Conclusion

This study identified three latent profiles of self-control, SA, and negative attentional bias among college students, with the profiles showing different associations with insomnia symptoms. Network analysis further highlighted SA1 (withdrawal behavior) and APNI-N (negative attention) as important features in the complex relationships among self-control, SA, negative attentional bias, and insomnia symptoms. These findings provide a more comprehensive understanding of the joint patterns and interconnected psychological and behavioral characteristics associated with insomnia symptoms among college students. These findings may provide useful implications for educators in identifying students with potentially vulnerable psychological and behavioral profiles and in developing more targeted health education and support strategies.

## Data Availability

The datasets generated during the current study are not publicly available due to ethical restrictions and participant privacy considerations but are available from the corresponding author upon reasonable request. Requests to access the datasets should be directed to Xinhong Zhu, zxh88@hbucm.edu.cn.
