# Supplementary material for: Self-control, smartphone addiction, negative attentional bias and insomnia symptoms among college students: a latent profile and network analysis
Source: Front Psychol. 2026 Oct 14;17:1976921. doi: 10.3389/fpsyg.2026.1976921 (PMC13713827; doi:10.3389/fpsyg.2026.1976921)
Supplement: Supplementary file 1 [file Supplementary_file_1.docx]

Supplementary Material

Table S1 Multicollinearity diagnostics for variables included in the regression model

| Variable | GVIF | Df | GVIF^^(1/(2×Df))^ |
| --- | --- | --- | --- |
| Profile | 1.09 | 2 | 1.02 |
| Gender | 1.19 | 1 | 1.09 |
| Grade | 1.49 | 2 | 1.10 |
| Major | 1.32 | 5 | 1.03 |
| Place of residence | 1.13 | 1 | 1.06 |
| Bedtime | 1.19 | 3 | 1.03 |
| Only child | 1.14 | 1 | 1.07 |
| Number of dormitory residents | 1.92 | 5 | 1.07 |

Note: GVIF = Generalized Variance Inflation Factor; Df = Degrees of Freedom; GVIF^^(1/(2×Df))^ represents the adjusted GVIF for variables with more than one degree of freedom.

**Table S2** Average Posterior Probability Matrices

| Assigned Class | Class 1 | Class 2 | Class 3 | Sample size |
| --- | --- | --- | --- | --- |
| 1 | **0.944** | 0.026 | 0.029 | 1201 |
| 2 | 0.140 | **0.860** | 0.000 | 173 |
| 3 | 0.092 | 0.000 | **0.908** | 223 |

Note: Participants were assigned to their most likely latent profile based on the highest posterior probability of profile membership. Values represent the average posterior probabilities of membership in each latent profile among participants assigned to a given profile. Bold values indicate the average posterior probability corresponding to the assigned profile. Sample size indicates the number of participants assigned to each profile.

**Table S3** Characteristics of the three latent profiles

|  | N (%) | Self-control score | Smartphone addiction score | Negative attention score |
| --- | --- | --- | --- | --- |
| Class 1 | 1201 (75.20) | 57.9 | 60.5 | 35.9 |
| Class 2 | 173 (10.83) | 44.3 | 87.8 | 45.5 |
| Class 3 | 223 (13.96) | 73.3 | 34.2 | 23.1 |

Note: N = number of participants; Self-control score, smartphone addiction score, and negative attention score represent the mean scores of the corresponding measures. Class 1: “Moderate Self-Control–Moderate SA and Negative Attention Profile”; Class 2: “Low Self-Control–High SA and Negative Attention Profile”; Class 3: “High Self-Control–Low SA and Negative Attention Profile”.

**Figure S1.** Network structures of self-control, smartphone addiction, negative attention, and insomnia symptoms across three latent profiles. Note: (A) Class 1; (B) Class 2; (C) Class 3. Orange edges indicate positive associations, whereas blue edges indicate negative associations. Class 1: “Moderate Self-Control–Moderate SA and Negative Attention Profile”; Class 2: “Low Self-Control–High SA and Negative Attention Profile”; Class 3: “High Self-Control–Low SA and Negative Attention Profile”; SCS, self-control scale; SA, smartphone addiction; APNI_N, negative attention; AIS, Athens Insomnia Scale.

**Figure S2.** Bootstrapped difference test for EI of self-control, smartphone addiction, negative attention, and insomnia symptoms. (A) Total sample; (B) Class 1; (C) Class 2; (D) Class 3. Note: Gray boxes indicate that there are no significant differences between nodes’ expected influences, while black boxes indicate that there are significant differences between nodes’ expected influences. The numbers in the white boxes (i.e., diagonal lines) indicate the numerical values of the nodes’ expected influences. Class 1: “Moderate Self-Control–Moderate SA and Negative Attention Profile”; Class 2: “Low Self-Control–High SA and Negative Attention Profile”; Class 3: “High Self-Control–Low SA and Negative Attention Profile”; SCS, self-control scale; SA, smartphone addiction; APNI_N, negative attention; AIS, Athens Insomnia Scale.

**Figure S3.** Standardized strength and expected influence centrality across three latent profiles. Note: (A) strength centrality; (B) expected influence centrality. Strength centrality and expected influence are presented as z-scores. Higher values indicate greater centrality within the estimated network. Class 1: “Moderate Self-Control–Moderate SA and Negative Attention Profile”; Class 2: “Low Self-Control–High SA and Negative Attention Profile”; Class 3: “High Self-Control–Low SA and Negative Attention Profile”; SCS, self-control scale; SA, smartphone addiction; APNI_N, negative attention; AIS, Athens Insomnia Scale.

**Figure S4.** Bootstrapped difference test for bridge strength of self-control, smartphone addiction, negative attention, and insomnia symptoms. (A) Total sample; (B) Class 1; (C) Class 2; (D) Class 3. Note: Gray boxes indicate that there are no significant differences between nodesʹ bridge strength, while black boxes indicate that there are significant differences between nodesʹ bridge strength. The numbers in the white boxes (i.e., diagonal lines) indicate the numerical values of the nodesʹ bridge strength. Class 1: “Moderate Self-Control–Moderate SA and Negative Attention Profile”; Class 2: “Low Self-Control–High SA and Negative Attention Profile”; Class 3: “High Self-Control–Low SA and Negative Attention Profile”; SCS, self-control scale; SA, smartphone addiction; APNI_N, negative attention; AIS, Athens Insomnia Scale.

**Figure S5.** Bootstrapped confidence intervals of edge weights. (A) Total sample; (B) Class 1; (C) Class 2; (D) Class 3. Note: The red line indicates the edge estimated in the sample, and the black dot indicates the bootstrap mean for that edge. Gray areas represent 95% bootstrap confidence intervals for the edge, with narrower gray lines indicating more accurate estimates of the edge. Class 1: “Moderate Self-Control–Moderate SA and Negative Attention Profile”; Class 2: “Low Self-Control–High SA and Negative Attention Profile”; Class 3: “High Self-Control–Low SA and Negative Attention Profile”; SCS, self-control scale; SA, smartphone addiction; APNI_N, negative attention; AIS, Athens Insomnia Scale.

**Figure S6.** Stability of the expected influence and bridge strength of nodes. (A) Total sample; (B) Class 1; (C) Class 2; (D) Class 3. Note: The x-axis represents the percentage of the original sample size used in each step. The y-axis represents the average correlation between the centrality indices in the original network and the centrality indices that were re-estimated after excluding progressively increasing percentages of samples. The line indicates the correlation between the expected influence and bridging strength. Class 1: “Moderate Self-Control–Moderate SA and Negative Attention Profile”; Class 2: “Low Self-Control–High SA and Negative Attention Profile”; Class 3: “High Self-Control–Low SA and Negative Attention Profile”

**Table S4** Comparison of Network Structures Across Different Profiles

| Test Type | Statistic | Class 1 vs. Class 2 | Class 1 vs. Class 3 | Class 2 vs. Class 3 |
| --- | --- | --- | --- | --- |
| Omnibus test of Network structure invariance | *M* | 0.199 | 0.156 | 0.206 |
|  | unadjusted *P* values | 0.173 | 0.468 | 0.603 |
|  | FDR-corrected *P* values | 0.518 | 0.603 | 0.603 |
| Global strength invariance test | Global strength | 4.34 vs. 4.91 | 4.34 vs. 3.53 | 4.91 vs. 3.53 |
|  | *S* | 0.567 | 0.809 | 1.376 |
|  | unadjusted *P* values | 0.638 | 0.176 | 0.038 |
|  | FDR-corrected *P* values | 0.638 | 0.264 | 0.114 |

Notes: Class 1: “Moderate Self-Control–Moderate SA and Negative Attention Profile”; Class 2: “Low Self-Control–High SA and Negative Attention Profile”; Class 3: “High Self-Control–Low SA and Negative Attention Profile”; FDR, false discovery rate. *M* represents the maximum difference in edge weights between two networks; *S* represents the difference in global strength. *P* values were adjusted for multiple pairwise comparisons using the Benjamini–Hochberg false discovery rate procedure.
